# Near-field engineering of Fano resonances in a plasmonic assembly for maximizing CARS enhancements

**DOI:** 10.1038/srep20777

**Published:** 2016-02-10

**Authors:** Jinna He, Chunzhen Fan, Pei Ding, Shuangmei Zhu, Erjun Liang

**Affiliations:** 1 School of Physical Science & Engineering and Key Laboratory of Materials Physics of Ministry of Education of China, Zhengzhou University, Zhengzhou 450052, China; 2 College of Electric and Information Engineering, Pingdingshan University, Pingdingshan, 467000, China; 3 Department of Mathematics & Physics, Zhengzhou Institute of Aeronautical Industry Management, Zhengzhou 450015, China

## Abstract

Surface enhanced coherent anti-Stokes Raman scattering (SECARS) is a sensitive tool and promising for single molecular detection and chemical selective imaging. However, the enhancement factors (EF) were only 10~100 for colloidal silver and gold nanoparticles usually used as SECARS substrates. In this paper, we present a design of SECARS substrate consisting of three asymmetric gold disks and strategies for maximizing the EF by engineering near-field properties of the plasmonic Fano nanoassembly. It is found that the *E*-field “hot spots” corresponding to three different frequencies involved in SECARS process can be brought to the same spatial locations by tuning incident orientations, giving rise to highly confined SECARS “hot spots” with the EF reaching single-molecule sensitivity. Besides, an even higher EF of SECARS is achieved by introducing double Fano resonances in this plasmonic nanoassembly via further enlarging the sizes of the constituent disks. These findings put an important step forward to the plasmonic substrate design for SECARS as well as for other nonlinear optical processes.

Coherent anti-Stokes Raman scattering (CARS), a well-known tool in multiphoton imaging and nonlinear spectroscopy, has been widely used in molecular identification and vibrational bioimaging since the late nineties^1^. In CARS process, the incident beams with two different frequencies *ω*_p_ and *ω*_s_ interact coherently through the third-order susceptibility (*χ*^(3)^) of the material, thereby generating a spectrally separated, blue-shifted beam at the anti-Stokes frequency of *ω*_as_ = 2*ω*_p_−*ω*_s_. Similar to surface enhanced Raman scattering (SERS), one of methods to improve CARS spectral sensitivity is to employ surface plasmons generated on metallic nanoparticles, which is generally named as surface enhanced CARS (SECARS)^2^,^3^. Unfortunately, though many efforts have been made since the first experimental observation of SECARS^2^, the theoretically predicted enhancement factor (EF) of ~10^12^ for SECARS^3^ has never been realized experimentally over the past two decades, unlike that for SERS. In previous work^4^,^5^,^6^,^7^, colloidal silver or gold nanoparticles were usually employed as plasmonic SECARS substrates and only a 10–100 times signal enhancement over conventional CARS was observed.

According to the theoretical analysis provided by Chew *et al.*^3^, the scattered field at the anti-Stokes frequency in SECARS process is expressed as:


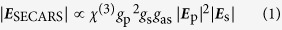


where *χ*^(3)^ is the third-order susceptibility of probe molecules; and ***E***_p_ and ***E***_s_ represent the incident pumping and Stokes fields with the respective frequencies of *ω*_p_ and *ω*_s_; and *g*_p_, *g*_s_ and *g*_as_ represent the EF of the pumping (***E***_p_), Stokes (***E***_s_) and anti-Stokes (***E***_SECARs_) fields induced by surface plasmons generated on plasmonic substrates, respectively. Therefore, the electromagnetic EF for SECARS signal is given by *G* = *g*_p_^4^*g*_s_^2^*g*_as_^2^, which should be much higher than that for SERS (*G*_SERS_ = *g*_p_^2^*g*_s_^2^) due to its higher-order dependence on incidence power.

Why is the observed EF for SECARS quite low and even much lower than that for SERS for colloidal silver or gold nanoparticles? The SERS is a two photon process while SECARS is a four photon process. From Eq. (1), an optimum plasmonic SECARS substrate requires (i) high simultaneous ***E***-field enhancement for the pumping, Stokes and anti-Stokes photons at the corresponding frequencies and (ii) all the enhanced ***E***-fields at three different frequencies, commonly known as “hot spots”, being spatially localized at the same position. Nevertheless, this is not possible generally because surface plasmon resonances with different frequencies originate from different plasmon modes and have usually different polarization states with different distributions/locations in *E*-field enhancement region. For colloidal nanoparticles or nanoassembly, the four photons in SECARS process with three different frequencies of *ω*_p_, *ω*_s_, and *ω*_as_ could not generally be enhanced simultaneously due to its single-band plasmon resonance or multi-band resonances but with different “hot spot” locations. It is obvious that the design for plasmonic SECARS substrates has more stringent requirements than that for SERS substrates.

Very recently, it was proposed by Halas *et al.* to apply plasmonic Fano nanostructures as SECARS substrates due to its unique light harvesting abilities^8^, wherein the spectral feature of Fano resonance with one dip and two shoulders, arising from the interference between bright and dark plasmon modes^9^,^10^,^11^,^12^,^13^,^14^, could just match the wavelengths of the pumping, Stokes and anti-Stokes beams in SECARS process. In this case, all photons with three frequencies are in resonance simultaneously, but not in the same spatial locations due to different ***E***-field polarizations in bright and dark resonant elements. This indicates that the “hot spots” of the Fano substrate at three frequencies of SECARS contribute individually to the SECARS EF. Therefore, to design or engineer plasmonic Fano structures with “hot spots” occurring in the same spatial locations at different spectral positions should be critical and challenging for SECARS applications.

In this work, we explore how to bring the ***E***-field “hot spots” for the photons involved in SECARS process with three different frequencies to the same spatial position in a plasmonic Fano nanoassembly consisting of three different-sized gold disks. It is found that the ***E***-field “hot spots” of the trimer pointing at a specific spectral position of Fano resonance could be tuned by varying the incident angle of excitation light, and hence the pumping, Stokes and anti-Stokes photons are in resonance in the same spatial position with the supported trimer substrate, leading to the highly confined SECARS “hot spots” with the EF higher than that of no near-field optimization. Besides, double Fano resonances are also demonstrated in this trimer by further enlarging the sizes of the constituent disks, which produces an even higher SECARS enhancement. These findings put an important step forward to the plasmonic substrate design for SECARS as well as for other nonlinear optical processes, such as four wave mixing and stimulated Raman scattering, *etc.*, in which multi-photons with different frequencies are involved and multi-frequency resonance and “hot spots” spatially overlapping are required.

## Results

### Fano-resonant structures

**Figure 1:**
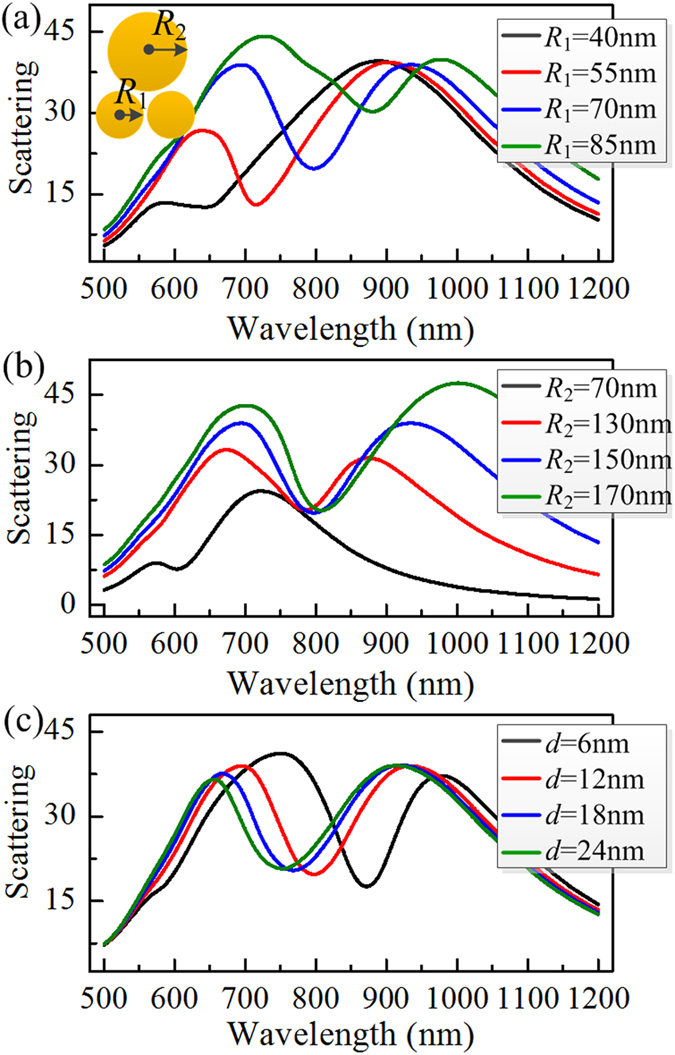
The dependence of scattering spectrum of an individual trimer on geometrical parameters. (**a**) Varying the radius of the lower dimer disks (*R*_1_) with fixed *R*_2_ = 150 nm and *d* = 12 nm. (**b**) Varying the radius of the upper disk (*R*_2_) with fixed *R*_1_ = 70 nm and *d* = 12 nm. (**c**) Varying the inter-disk gap distance (**d**) with fixed *R*_1_ = 70 nm and *R*_2_ = 150 nm. Here the trimer is excited by normal incident plane wave with ***E***-polarization parallel to the line connecting the centers of dimer disks. The inset displays a sketch of a trimer structure.

Plasmonic Fano resonance, an analogy of quantum interference between the continuums with the discrete states in atomic physics, has been demonstrated recently in a series of plasmonic systems depending on various excitation ways. For example, both the dolmen-type slab structures^15^,^16^ and nonconcentric ring/disk cavity^9^,^17^ exhibit Fano resonances via the destructive interference between a superradiant (bright) and a subradiant (dark) plasmon mode supported by different sub-elements of the nanostructures. Plasmonic nanoassembly consisting of three^18^,^19^, four nanoparticles^20^ and even larger aggregates^21^ also exhibit sharp Fano interference due to the linear combinations of the plasmon modes in each constituted nanoparticles with sufficiently small interparticle separations. In addition, Fano resonance can be also observed in heterodimers with the interactions between a continuum interband transition absorption and a dipolar resonance^22^ or in various array or grating structures with the interactions between the narrow diffractive resonance and broad plasmon modes^23^,^24^. Here a plasmonic assembly consisting of three gold disks with different sizes^19^ (See the inset in Fig. 1(a)), which supports Fano resonance by the hybridization of electric (superradiant) and magnetic (subradiant) plasmon modes^19^,^25^,^26^, is chosen owing to relatively simple configuration and strong ***E***-field "hot spots" occurring at the gap region.

### Spectral tunabilty of Fano resonance

In this disk trimer nanoassembly, the interference between a superradiant electric mode, where the dipolar plasmons inside three disks oscillate in-phase, and a subradiant coil-type magnetic mode, which is formed by a phase retardation between the upper large disk and the lower small disk dimer, generates Fano resonance that can be tuned to various wavelengths by varying geometrical parameters of trimer, as shown in Fig. 1. From Fig. 1(a), the Fano dip moves to red clearly with *R*_1_ increasing from 40 to 85 nm, in accordance to the shift of the coil-type magnetic resonance. As changing *R*_2_ from 130 to 170 nm, the Fano dip essentially retains its spectral position at or near 800 nm, while the superradiant shoulder at lower-energy side of the Fano minimum, corresponding to electric mode, is shifted significantly (See Fig. 1(b)). It is also observed that in symmetric equilateral trimer with *R*_2_ = 70 nm, the Fano dip is suppressed and only a broad-band Lorenz resonance arising from the electric mode is left around at λ = 720 nm due to the disappearance of the magnetic mode. Figure 1(c) displays the spectral dependence on the inter-disk gap distance (*d*) of trimer. It is seen that the Fano dip blue shifts clearly when the gap spacing is increased gradually. This is attributed to the decreased coupling between the disks within the trimer^21^,^27^, which leads to a spectral blue shift of the subradiant magnetic mode. In these calculations, the trimer disk’s thickness is always kept constant at *h* = 40 nm, which has little influence on the scattering spectrum of the trimer.

### SECARS enhancement

**Figure 2:**
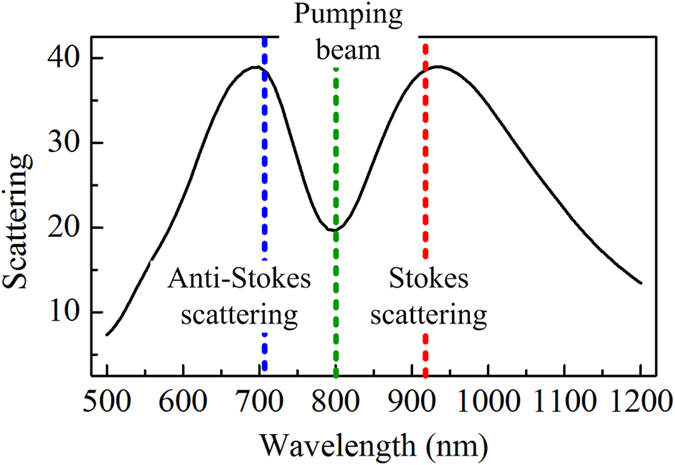
Scattering spectrum of the trimer with optimized geometrical parameters (*R*_1_ = 70 nm, *R*_2_ = 150 nm and *d* = 12 nm) for enhancing the *p*-MA 1580 cm^−1^ mode with an 800 nm pumping laser. Here the dip and two shoulders of Fano resonance are just matched to the wavelengths of pumping (green, 800 nm), Stokes (red, 916 nm) and anti-Stokes (blue, 710 nm) beams, respectively.

To evaluate SECARS enhancements of the plasmonic Fano trimer nanoassembly, we next calculate the distribution pattern and magnitude of the SECARS EF (*G* = *g*_p_^4^*g*_s_^2^*g*_as_^2^) for a Raman mode of *para*-mercaptoaniline (*p*-MA) molecules at 1580 cm^−1^. If assuming with an 800 nm wavelength pumping laser, the corresponding Stokes and anti- Stokes wavelengths are 916 and 710 nm, respectively. By optimizing geometrical parameters of the trimer, we tune the Fano dip just corresponding to the wavelength of pumping laser (green, 800 nm), and the shoulder to the red and blue of Fano minimum corresponding to the wavelengths of the Stokes (red, 916 nm) and anti-Stokes (blue, 710 nm) (See Fig. 2). This should be most advantageous for SECARS enhancements because the Fano dip is very close to the maximum in the near-field enhancement spectrum^28^ and meanwhile the superradiant shoulder could maximize the coupling of anti-Stokes light to the far field, as demonstrated in previous work^8^,^12^.

**Figure 3:**
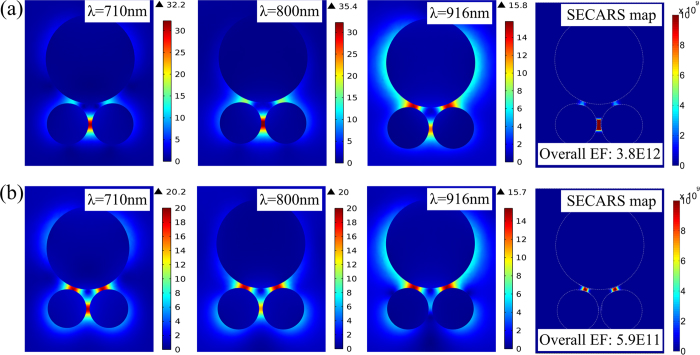
The near-field amplitude (| *E*/*E*_0_|) distributions of the trimer at the wavelengths of pumping (800 nm), Stokes (916 nm) and anti-Stokes (710 nm), and the corresponding SECARS map (*G* = *g*_p_^4^*g*_s_^2^*g*_as_^2^) for (a) normal, and (b) 30^0^-oblique incidence plane wave excitations. Here the ***E***-field amplitude (|***E***/***E***_0_|) at three characteristic wavelengths is evaluated in a plane 1 nm above the top surface of trimer. The overall SECARS EF, by integrating the SECARS map over the top surface of the trimer, is given in lower right corner of each SECARS map.

Figure 3(a) gives the spatial distributions of near-field amplitude (|***E***/***E***_0_|) of the trimer at three characteristic wavelengths and calculates the corresponding SECARS EF distribution. It can be seen that at the wavelengths of the pumping (800 nm) and anti-Stokes (710 nm), the “hot spot” of ***E***-fields occurs at the lower gap between the dimer disks, whereas at the Stokes wavelength (916 nm), the “hot spot” at the lower gap is suppressed and two new “hot spots” are visible at the upper gaps of trimer. The distribution of SECARS EF has a complex spatial dependence, or a “map” of the SECARS response across the trimer since the local ***E***-field intensity is a function of spatial coordinate. From the SECARS map in Fig. 3(a), it can be observed clearly that the “hot spot” with large SECARS EF mainly occurs at the lower gap, which is of greater magnitude than that at the upper gaps. For providing an overall relative enhancement, the overall SECARS EF of the trimer substrate is calculated by integrating the SECARS map over the top surface of the trimer, which is presented in the lower right corner of the panel. For comparison, we also calculated the overall SECARS EF of the symmetric equilateral trimer (not shown), which is ~10 times smaller than that of Fano resonance in this trimer. This indicates that plasmonic Fano structure is a more promising choice for SECARS substrate due to its stronger light harvesting abilities.

As discussed previously, the coherent resonances in the same spatial location for distinct resonant modes, forming the “mixed frequency coherent mode”^29^, should be highly desirable for SECARS substrates. However, this is generally impossible for simple plasmonic structures. As shown in Fig. 3(a), the ***E***-field “hot spots” of the trimer at three characteristic wavelengths occur in different spatial positions. When the excitation orientation of plane wave is changed from the normal to 30^0^-oblique incidence, it is interesting to find that the spatial locations of the “hot spots” are modified significantly even at the same wavelengths. From Fig. 3(b), it is found that the “hot spots” are transferred efficiently from the bottom to the upper gaps at the wavelengths of pumping (800 nm) and anti-Stokes (710 nm), while the spatial localization at the Stokes wavelength (916 nm) is kept nearly unchanged, still located at the upper gaps. As a result of the same spatial localizations at the three wavelengths, two tightly confined SECARS “hot spots” are generated. From the SECARS map in Fig. 3(b), the maximum EF indeed occurs at the upper gaps. Because three ***E***-field amplitudes in Fig. 3(b) are lower than that in Fig. 3(a), the overall EF of ~5.9 × 10^11^ is slightly smaller than the value of ~3.8 × 10^12^.

Significant engineering of the dynamics of the trimer’s near-field spatial localizations at a specific wavelength via the variation of illumination orientation should be attributed to the destructive or constructive interference between the near-fields arising from the electric and magnetic modes^30^. The ***E***-fields at the bottom gap are dominated by the contribution from the magnetic mode, whereas the ***E***-fields at the upper two gaps have significant contributions from both electric and magnetic modes. Under normal incidence, the ***E***-fields at the upper gaps are strongly suppressed due to the destructive interference between the near-fields of the electric and magnetic modes, and thus the strongest ***E***-field enhancements appears at the bottom gap of the trimer. With a 30^0^ shift of excitation direction, the ***E***-fields at the upper gaps are significantly enhanced as a result of their constructive interference. Engineering the interference and interaction between the plasmon modes enables a powerful control over the near-field properties of complex nanostructures.

### Dependence of SECARS enhancement on the excitation angle

**Figure 4:**
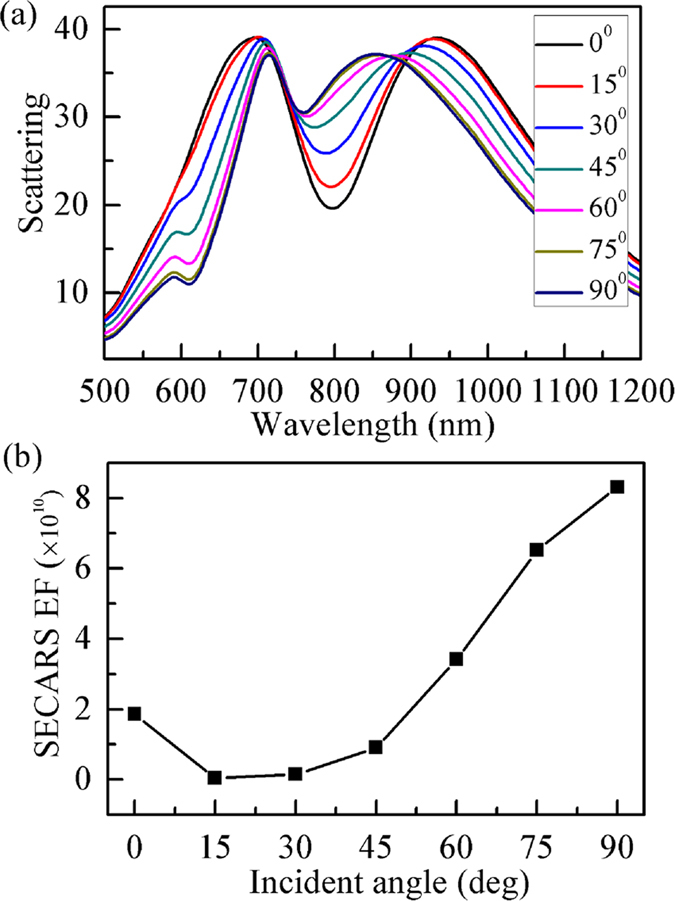
(**a**) Scattering spectra of the trimer at various incident angles from 0^0^ to 90^0^ with an increment of 15^0^. (**b**) The maximum value in each SECARS map for various incident angles is extracted. Here the SECARS map is calculated for the ***E***-field amplitude at three characteristic wavelengths corresponding to the spectral positions of the Fano minimum and two shoulders.

Here we examine how the excitation angle of plane wave affects the scattering spectrum and SECARS EF of the trimer substrate. Figure 4(a) displays the scattering spectrum of the trimer verse various excitation angles ranging from 0^0^ to 90^0^. It is found that the Fano dip gradually weakens and blue shift generates when the incident angle is increased. As the incident angle becomes more grazing, the magnetic mode, which is excited indirectly under normal incidence, can couple directly to the excitation light by the magnetic field component along the trimer normal. Therefore, the coupling between the electric and magnetic modes is altered with the incident angle due to varying amplitude and phase on the magnetic mode, resulting in such a change of the line shape of Fano resonance.

The maximum value in each SECARS map for various incident angles is extracted in Fig. 4(b), where three ***E***-field intensities are evaluated still in a plane 1 nm above the top surface of the trimer substrate. From Fig. 4(b), it is observed that the SECARS EF at first decreases slightly for the incident angle varying from 0^0^ to 15^0^. This mainly lies in the weakened Fano resonance by the oblique incidence, which leads to the decrease of three ***E***-field amplitudes with a resulting reduction of SECARS EF. Subsequently, once the incident angle exceeding 15^0^, the coupling between the near-fields of the electric and magnetic modes is changed from the destructive to constructive interference. Thus, three ***E***-field amplitudes at the upper gaps become stronger and stronger with the increase of the incident angle, resulting in an obvious and continuous increase of the EF. Finally, the highest EF, up to ~8.31 × 10^10^, is achieved at the incident angle of 90^0^. This indicates that the SECARS signal can be enhanced by more than ~10 orders of magnitude relative to standard CARS, reaching single-molecule detection sensitivity^31^,^32^. Furthermore, the SECARS EF is ~4.47 times of no near-field optimization, demonstrating that the near-field engineering of plasmonic Fano substrates has significance for SECARS applications.

### Double Fano resonances

**Figure 5:**
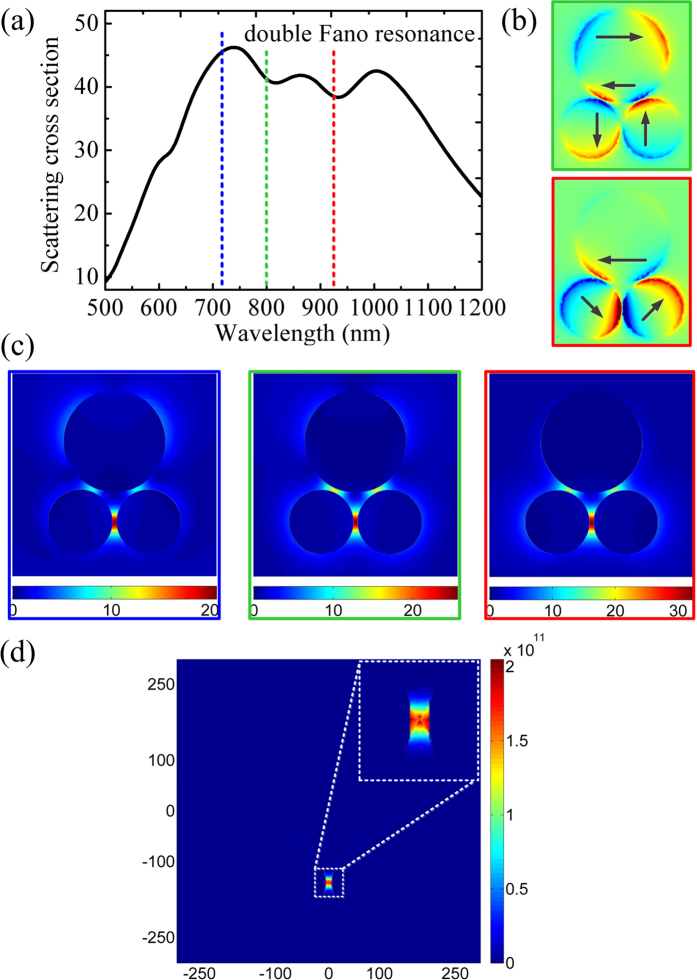
(**a**) Scattering spectrum of the trimer (*R*_1_ = 95 nm, *R*_2_ = 150 nm, *d* = 12 nm) at normal incidence of plane wave. Blue dashed line: the anti-Stokes scattering (710 nm); green dashed line: the pumping laser (800 nm); red dashed line: the Stokes scattering (916 nm). (**b**) Illustration of spatial distributions of *E*_z_ component on the top surface of the trimer excited at 800 nm pumping (top) and 916 nm Stokes (bottom) wavelengths, corresponding to two subradiant modes. (**c**) Near-field (|***E***/***E***_0_|) distributions in a plane 1 nm above the top surface of trimer at the anti-Stokes (left), pumping (middle) and Stokes (right) frequencies. (**d**) The SECARS map (*G* = *g*_p_^4^*g*_s_^2^*g*_as_^2^) in (**c**). The maximum EF of ~2.08 × 10^11^ is achieved at the center of the gap between the lower dimer disks.

Figure 5(a) shows the scattering spectrum of the trimer with *R*_1_= 95 nm, *R*_2_ = 150 nm and *d* = 12 nm under normal incidence of plane wave. It is found that by further increasing the size of the dimer disks, two pronounced Fano resonances appear with the narrow minimum centered at the wavelengths of about 800 and 916 nm on top of a far broader superradiant resonance, respectively. The origin of double Fano resonances can be understood by examining the *z*-component of electric fields (*E*_z_) at their respective Fano dip spectral position (See Fig. 5(b)). For the Fano resonance at 800 nm, the dipole of the lower, left disk oscillates out of phase with respect to the lower, right disk, and simultaneously a clear quadrupole pattern is observed inside the upper large disk, indicating a small net dipole moment for the resonant mode and its subradiant feature. As for the Fano resonance at 916 nm, a planar coil-type current oscillation forming a magnetic resonance can be observed in the trimer, as denoted by black lines with arrow. These two subradiant modes resonating at their respective spectral positions are superimposed on the spectral envelope of the broad electric (superradiant) resonance, thereby producing two pronounced Fano dips in scattering spectrum due to the destructive interference between subradiant and superradiant modes. The Fano resonance at 800 nm is formed as a new one depending on the excitation of high-order plasmon mode inside the upper large disk, while another one at 916 nm is a result of the red shift of the original Fano resonance due to the increase of the size of the lower dimer disks. Obviously, the two Fano resonances could be independently tuned to various wavelengths by varying the size of the constituent disks.

It should be most advantageous for plasmonic SECARS substrates to minimize the losses at both excitation wavelengths while maximizing the far-field coupling at the wavelength of anti-Stokes emission^33^. The reduction of the scattered light at the wavelength corresponding to the Fano dip maximizes energy coupling into the nanostructure, thereby producing highly localized, intense field enhancements. The superradiant shoulder acts like an optical antenna, maximizing the propagation of the anti-Stokes emission to the far field. Through optimizing the trimer’s geometrical parameters, we tune the two Fano dips just corresponding to the wavelengths of the pumping (green, 800 nm) and Stokes (red, 916 nm), and the shoulder to the blue of the first Fano minimum corresponding to the anti-Stokes wavelength (blue, 710 nm) in the SECARS spectrum for the 1580 cm^−1^ Raman mode of *p*-MA molecules, as shown in Fig. 5(a). The ***E***-field amplitude (|***E***/***E***_0_|) distributions at the wavelengths of anti-Stokes (blue line), pumping (green line), and Stokes (red line), in a plane 1 nm above the top surface of the trimer, are shown in Fig. 5(c), respectively. The corresponding SECARS map is given in Fig. 5(d). Obviously, the strong ***E***-field localizations in the same spatial location at three wavelengths leads to a tightly confined SECARS “hot spot” occurring at the gap between the lower dimer disks (See the inset in Fig. 5(d)), with the EF up to ~2.08 × 10^11^. This value is roughly ~11 times as large as that of the trimer with single Fano resonance (~1.86 × 10^10^), indicating that plasmonic structure with double Fano resonances should be a more promising choice for SECARS substrates as well as other nonlinear optical process.

## Discussion

Actually, the SECARS enhancements could be further amplified by decreasing the inter-disk gap distance of the trimer. When the gap distance is decreased, the amplitude of ***E***-field “hot spots” at the gaps will be enhanced dramatically owing to the increased near-field coupling between the constituent disks^34^,^35^, thereby resulting in a significant increase of SECARS EF. We thus calculate the SECARS map of the trimer with *R*_1_ = 70 nm, *R*_2_ = 150 nm and the gap distance of 6 nm, still for the 1580 cm^−1^ Raman mode with the 800 nm wavelength pumping laser (not shown). It is found that the maximum SECARS EF reaches ~1.10 × 10^13^ inside the lower gap region, increased dramatically relative to the value occurring in the trimer with the 12 nm gap distance.

In summary, we analyzed the requirements for optimizing plasmonic SECARS substrates and explored strategies to bring the ***E***-field “hot spots” for the photons involved in SECARS process with different frequencies to the same spatial position in a plasmonic Fano assembly consisting of three asymmetric disks. It is found that (i) the ***E***-field “hot spots” of the trimer pointing at a specific spectral position of Fano resonance can be tuned actively by changing the excitation orientation of plane wave, yielding highly confined SECARS “hot spots” with the EF reaching single-molecules detection sensitivity; (ii) the plasmonic trimer assembly supporting double Fano resonances provides an efficient method for generating the “mixed frequency coherent mode”. These findings put an important step forward to the plasmonic substrate design for SECARS as well as for other nonlinear optical processes such as four wave mixing and stimulated Raman scattering, *etc.*, in which multi-photons with different frequencies are involved and multi-band resonances and “hot spots” spatially overlapping are required.

### Methods

Numerical simulations were carried out using the finite element method (FEM) with the implement of COMSOL Multiphysics, where Perfect Matched layers (PML) were used to avoid spurious reflections at the surrounding boundaries of an isolated plasmonic trimer, and the scattering cross section was computed in the framework of the scattered formulation. In all cases, for the excitation of Fano resonance, the trimer structure was illuminated by plane wave with its *E*-polarization along the line connecting the centers of the lower dimer disks. The permittivity values of gold were taken from the experimental data given by Johnson and Christy^36^. The surrounding dielectric environment was assumed to be air with the refractive index of *n* = 1 for our calculation simplification. Notice that the introduction of the dielectric substrates or probe molecules in practice does not modify the optical properties of the trimer structure, but only shift the Fano resonance to longer wavelengths accompanied with a slight increase in linewidth because of dielectric screening^37^,^38^. In addition, the calculated SECARS EF depends on the size of the FEM meshgrid, which is chosen to be always consistent in all calculations for providing a relatively fair comparison.

## Additional Information

**How to cite this article**: He, J. *et al.* Near-field engineering of Fano resonances in a plasmonic assembly for maximizing CARS enhancements. *Sci. Rep.* **6**, 20777; doi: 10.1038/srep20777 (2016).
